# The Risk of Osteoporotic Forearm Fractures in Postmenopausal Women in a Siberian Population Sample

**DOI:** 10.3390/jpm10030077

**Published:** 2020-07-31

**Authors:** Elena Mazurenko, Oksana Rymar, Liliya Shcherbakova, Ekaterina Mazdorova, Sofia Malyutina

**Affiliations:** 1 Research Institute of Internal and Preventive Medicine–Branch of the Institute of Cytology and Genetics, Siberian Branch of Russian Academy of Sciences, Novosibirsk 630089, Russia; orymar23@gmail.com (O.R.); 9584792@mail.ru (L.S.); mazdorova@mail.ru (E.M.); smalyutina@hotmail.com (S.M.); 2 Novosibirsk Research Institute of Traumatology and Orthopedics named after Ya. L. Tsivyan, Novosibirsk 630112, Russia

**Keywords:** osteoporosis, fracture, menopause, population, chronic non-communicable diseases, risk factors

## Abstract

The reduction in bone and muscle mass increases in menopausal women and poses a threat to the loss of *self*-*dependence* in the elderly. The aim of the study was to assess the frequency of osteoporotic forearm fractures (OFF) in postmenopausal women and to study their association with risk factors for chronic non-communicable diseases (NCD). The study was based on the Russian arm of the Health, Alcohol and Psychosocial Factors In Eastern Europe (HAPIEE) project (Novosibirsk). In a subsample of postmenopausal women aged 55–84 years old (n = 2005), we assessed the history of OFF during the last 3 years and risk factors for fracture and common NCD/. Cross-sectional associations between OFF history and potential determinants were analyzed using multivariable-adjusted logistic regression. A history of OFF in the last 3 years was found in 3.9% women. In a multivariable-adjusted model, the risk of OFF was directly associated with smoking in the past (OR = 2.23; 95% Cl 1.10–4.55), total cholesterol level higher than 200 mg/dL (OR = 1.98; 95% Cl 1.19–3.29), and it was inversely associated with body mass index (OR = 0.91; 95% Cl 0.86–0.96). In studied population sample of postmenopausal women the cross-sectional determinants of osteoporotic forearm fractures were smoking in the past and high total cholesterol value; body mass index protectively related to the risk of osteoporotic fractures. These findings might have implications for fracture prevention in postmenopausal women.

## 1. Introduction

The reduction in bone and muscle mass increases with age and poses a threat to the loss of *self*-*dependence* in the elderly and, specifically, during perimenopause. While the majority of women under the age of 50 years have normal bone mineral density (BMD), by the age of 80 years, 27% of them have osteopenia and 70% have osteoporotic BMD values when examining the thigh, lumbar spine or forearm [1]. According to the published data, the prevalence of osteoporotic forearm fractures (OFF) is the highest among fracture locations. At the age of about 50 years, Caucasian women have a lifetime risk of a wrist fracture about 16% [2]. Forearm fractures are clinically important outcomes from the perspective of morbidity, health care costs, and interruption of work [3]. In the Study of Osteoporotic Fractures, the elderly women with wrist fractures were almost 50% more likely to have a clinically important functional decline than those without fractures [4].

The risk of death within 5 years after an occurrence of OFF ranges from 12% among women aged 65 to 74 years to 43% for women aged 85 years and older [5]. Another study found that low BMD in the distal forearm, categorized as osteopenia and osteoporosis, was associated with increased mortality, and the association was only slightly attenuated by taking osteoporotic fractures into account [6]. This may suggest that low BMD is more powerful predictor of death than an increased risk of fracture [7].

For the Russian Federation, OFF is a serious problem, because most of these fractures occur during the cold season as a result of falling on the ice and this season might be from October to April. Referring the epidemiological study in 16 cities of Russia, the frequency of forearm fractures was 426/100,000 which exceeds the frequency of hip fracture by 3–7 times in men and 4–8 times in women, and it occurred significantly more in women [8,9]. Moreover, in such cities as Moscow, Tyumen, Khabarovsk and Yekaterinburg, women had a frequency of forearm fractures near 1200 per 100,000 and higher [10].

Forearm fractures may precede future secondary fractures. However, if a forearm fracture occurs in a patient with low BMD, this may result in even more severe fragility fractures such as femoral and vertebral fractures [11,12].

Dual-energy X-ray absorptiometry (DXA) screening is standard in the US (at the age of 65 years in women and age 70 in men, and in individuals over the age of 50 years who have suffered an adult fracture) [13], but, in the majority of other countries, population screening is not judged to be cost-effective and primary prevention is focused more on opportunistic case finding, triggered by the presence of clinical risk factors [14,15]. A recent randomized-controlled trial (the UK SCOOP) has investigated the effectiveness and cost-effectiveness of screening in older women in primary care for the prevention of fractures in seven centers where approximately 12,500 older women were randomized to either normal care or screening and subsequent treatment (based on the FRAX risk assessment tool). This study has demonstrated that this intervention leads to a 28% reduction in hip fracture risk [16]. As would be expected from this approach, the screening appeared to be the most effective in those at highest baseline fracture risk [17], and importantly, it was shown to be cost-effective [18,19]

Increased attention to OFF is important to identify women at increased risk for repeated future fractures and to apply preventive measures [20]. A high frequency of comorbidity of severe osteoporosis with NCD has been established [21]. Cardiovascular disease (CVD) is known to be associated with an increased risk of hip fractures [22]; similarly, it has been shown that low bone mass in women can be an independent predictor of CVD [23]. Diabetes mellitus (DM) is also a risk factor for hip fracture. Recent studies have shown that microstructural changes in bone tissue are more noticeable among people with diabetes with microvascular complications [24]. Commonly accepted risk factors for NCD probably contribute to the development of osteoporosis.

The aim of the study was to assess the frequency of osteoporotic forearm fractures in women aged over 55 years and to investigate the association between OFF and common risk factors for NCD.

## 2. Studied Population and Methods

### 2.1. Partisipants

The data came from the Russian arm of the Health, Alcohol, and Psychosocial Factors in Eastern Europe (HAPIEE) project. The random population sample (9360 men and women aged 45–69 years old) was examined in Novosibirsk (Russia) at baseline in 2003–2005 and re-examined twice. In 2015–2018, a sample of 3898 subjects was examined in the frame of the wave-3 (men, women aged 55–84 years). Current analysis was restricted to a random subset of women who gave answers to the question on history of fractures (n = 2005). Additionally, we excluded those with fractures that occurred more than 3 years ago who had no measurement of fasting blood glucose levels (FBG), and women in the reproductive period. The final sample for this analysis included 2005 women. The study was approved by the Ethics Committee of the Research Institute of Internal and Preventive Medicine NIITPM (Protocol, dated 12/26/2014).

### 2.2. Study Questionnaire

The program of examination of the HAPIEE project is published elsewhere [25] (http://www.ucl.ac.uk/easteurope/hapiee-cohort.htm). The data on fractures of the forearm in the last 3 years, the history of DM2, the duration of menopause in women, smoking and alcohol intake habits, physical functioning and other risk factors for CVD and NCD, and socio-demographic data were collected using the structured questionnaire.

The osteoporotic forearm fracture was defined as the history of fracture within the last 3 years which occurred when the subject fell from his own growth or spontaneously.

The smoking questionnaire included information about current smoking one or more cigarettes per day and smoking history in the past. A person was considered as a current smoker if he or she smokes at least one cigarette per day. Alcohol consumption was estimated using the Graduated Frequency Questionnaire (GFR). Physical activity was assessed using the Physical Functioning scale (PF10, subscale from SF-36). The value of PF10 scale distribution <75‰ was considered as low.

### 2.3. Anthropometry and Blood Pressure Measurement

Anthropometric measurements were performed including height, weight, waist and hip circumference. The height was measured standing, without outerwear and shoes, on a standard height meter with an accuracy of 0.5 cm. Body weight was determined without outerwear and shoes, on standard lever scales that underwent metrological control (with measurement accuracy 0.1 kg).

Waist hip ratio (WHR) and body mass index (BMI) was calculated by a common formula:

BMI (kg/m^2^) = body weight (kg)/height^2^ (m^2^) (1)

WHR (units) = waist circumference/hip circumference(2)

Blood pressure (BP) measurement was performed three times on the right hand in a sitting position after five minutes of rest at intervals of 2 min between measurements. The average value of three blood pressure measurements was used in the current analysis.

### 2.4. Biochemical Measurements

Blood sampling was performed after 8 h fasting. After centrifugation, the serum was stored in a low-temperature chamber (−70 °C). A biochemical blood test was performed at the Clinical Biochemistry Laboratory of NIITPM, standardized for Federal quality control at regular basis. The concentration of total cholesterol, high-density lipoprotein cholesterol (HDL-C), triglycerides (TG) and glucose in blood serum was carried out by the enzymatic method using commercial standard Biocon kits (Germany) on a KoneLab autoanalyzer (USA). The concentration of low-density lipoprotein cholesterol (LDL-C) was calculated by Fridewald formula. The conversion of serum glucose to plasma glucose (GP) was done by formula

GP (mmol/L) = −0.137 + 1.047 × serum glucose (mmol/L) (EASD, 2005).(3)

Diabetes mellitus, type 2 (DM2) was established by epidemiological criteria with FBG ≥7.0 mmol/L (WHO, 2003) and/or normoglycemia in patients with a medical history of established DM2.

### 2.5. Statistical Analysis

Statistical analysis was performed using the SPSS software package (v.13.0). The statistical significance of differences in average values was evaluated by Student’s criterion (t) for normally distributed characters. To determine the statistical significance of differences in qualitative characteristics, the Pearson method (χ^2^) was used. Comparison of two independent groups by quantitative characteristics with an abnormal distribution was made using the nonparametric Mann–Whitney criterion. The measures are presented as relative values (n, %), and average values (M ± SD), where M is the arithmetic mean value and SD is the standard deviation. Differences were considered statistically significant at *p*-value < 0.05. To assess the relationship between OFF in the last three years and common risk factors, the logistic regression method was used in age- and multivariable-adjusted models.

## 3. Results

### 3.1. Comparative Characteristics of the Studied Groups

In studied population sample of 2005 postmenopausal women the incidence of OFF comprised 3.9% (n = 71). The frequency of OFF in the last 3 years did not differ in women with DM2—3.0% and without DM2—4.3% (*p* = 0.218)

The subjects examined were split into two groups: those with a history of OFF and those without a history of OFF; comparative characteristics are presented in Table A1. Women with OFF had lower weight (*p* = 0.003), BMI (*p* = 0.001), waist circumference (*p* = 0.001), hip circumference (*p* = 0.013), WHR (*p* = 0.001), compared to those without OFF. The groups did not differ by age, BP values, lipid values, physical functioning, smoking, frequency of DM2 and education level.

### 3.2. The Relationship Between NCD Risk Factors and OFF by Logistic Regression Analysis

Logistic regression analysis was used to assess the association between the history of OFF in the last 3 years and common risk factors for NCD. Regression was performed in age-adjusted Model 1 and in multivariable–adjusted Model 2 and Model 3. The history of OFF in the last 3 years was used as a dependent variable. The tested factors were used as independent variables and include age (continuous measure; per 1 year), presence of DM2 (categorized as yes/no), smoking (categorized as never smoking/smoking in the past/present smoking), BMI (continuous measure; per 1 kg/m^2^), duration of menopause (categorized as ≥10 years/<10 years), physical performance (categorized in 2 categories having low pf10 < 75‰ and preserved pf10 value ≥ 75‰), total cholesterol value (categorized as total cholesterol ≥ 200 mg/dL and < 200 mg/dL) Model 2 was controlled for the following covariates: age, DM2, BMI, smoking, menopause, physical performance. Model 3 was controlled for the following covariates: age, DM2, smoking, physical performance, total cholesterol value.

**Figure A1:**
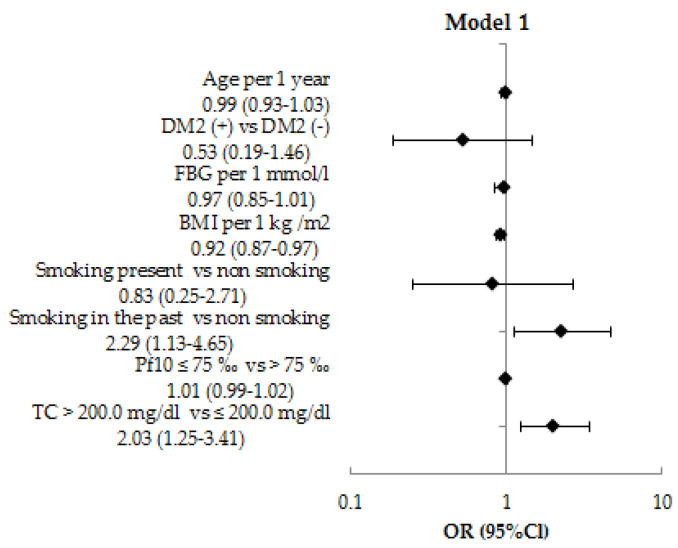

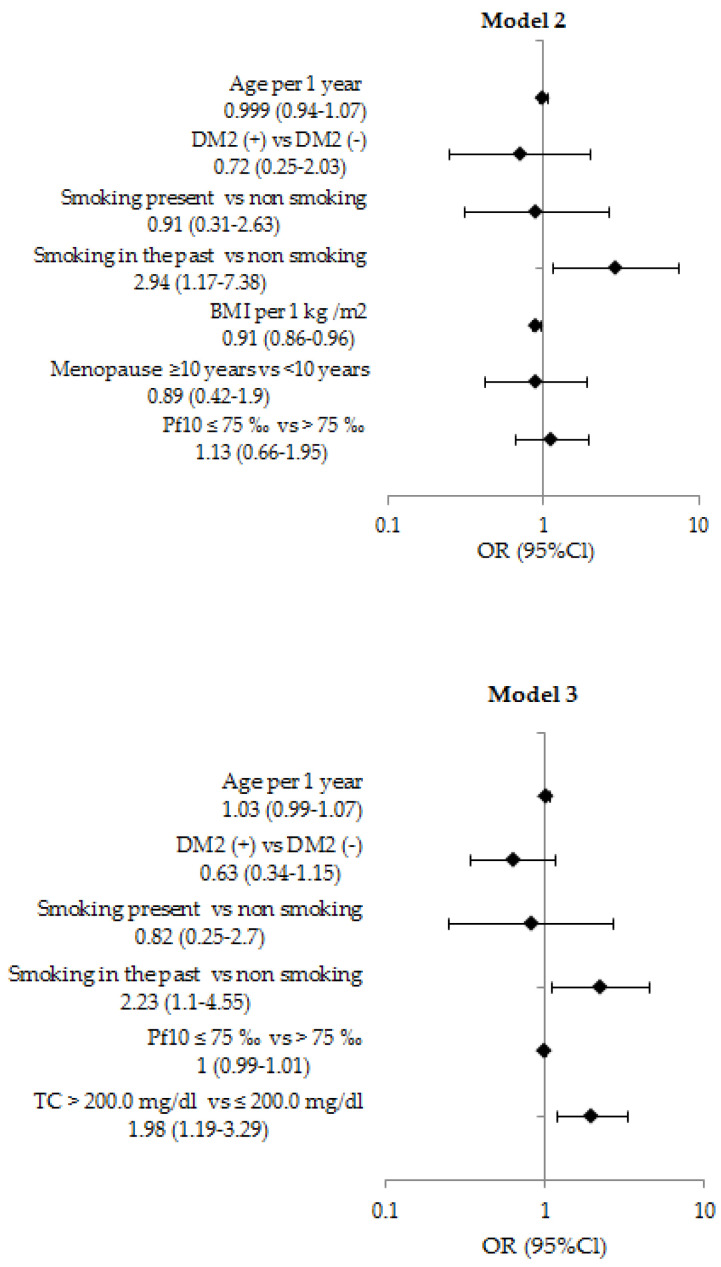
The results of a logistic regression analysis of the relationship OFF over the past 3 years and studied risk factors. Model 1—age-adjusted. Model 2 adjusted for age, DM2, BMI, smoking, duration of menopause, physical performance. Model 3 adjusted for age, DM2, smoking, physical performance, total cholesterol.

The results of logistic regression are presented on Figure A1. In age-adjusted analysis, the risk of OFF in postmenopausal women was positively associated with history of past smoking (OR = 2.29; 95% Cl 1.13–4.65), total cholesterol > 200 mg/dL (OR = 2.03; 95% Cl 1.25–3.41), and negatively associated with BMI (OR = 0.92; 95% Cl 0.87–0.97). In multivariable-adjusted analysis, these relationship remained significant and the risk of OFF was positively associated with history of past smoking (OR = 2.23; 95% Cl 1.10–4.55), elevated total cholesterol value > 200 mg/dL (OR=1.98; 95% Cl 1.19–3.29), and negatively associated with BMI (OR = 0.91; 95% Cl 0.86–0.96) regardless of other factors (Figure A1).

## 4. Discussion

In a studied population sample of postmenopausal women aged 55–80 years old, we revealed that substantial frequency of osteoporotic forearm fractures occurred during the last 3 years—3.9% OFF. In our cross-sectional analysis, the risk of OFF was associated with several risk factors of NCD, specifically, it was directly associated with past smoking, elevated total cholesterol and it was inversely related to BMI value independent of other factors.

The prevalence of OFF in the world is high and continues to increase. In the southern part of the Skane region (Sweden), the incidence of OFF is 278 cases per 100,000 persons. From 1999 to 2010, the total annual number of OFF in women increased from 1779 to 2323 [26]. Abrahamsen et al. [27], in a recent large-scale study in Denmark, showed that the incidence in women (530 per 100,000 persons) was slightly higher than in the Jerrhag D study [26].

We found the frequency of OFF occurred during the last 3 years among postmenopausal women in Novosibirsk of 3.9%. The results of our study confirm the high incidence of OFF in the Russian population. In the city of Pervouralsk (Russia), 586 OFF were registered over a two-year period, which amounted to an average of 540.7/100,000 persons, moreover, women experienced fractures five times more often than men (787.9/100,000 and 171.1/100,000 in women and men, respectively, *p* < 0.00001) [28]. According to study of Zavodsky B.V. et al. conducted in the period 2008–2014 in the Volgograd region, fractures of the radial bones dominated among all fractures both among those with osteoporosis and with normal bone mineral density (*p* < 0.0001) [29]. In the city of Ulan-Ude, the medical documentation of trauma centers was studied for the period of 2009–2011 and it was revealed that women most often underwent OFF—in 44.4% of cases among all fractures [30]. When calculating the rate per 100,000 persons of the corresponding nationality, it turned out that the Buryats suffered from complications of osteoporosis two times more often than Russians: 648.8 cases per 100,000 of inhabitants of the Mongoloid race and 323.6 cases per 100,000 of inhabitants of Slavic origin. However, women, regardless of nationality, most often suffered from the fractures of the distal forearm [30]. These published data [29,30] repeatedly focus on the fact that the BMD of the wrist might be underestimated in common medical practice. In our earlier study, we found that the BMD in the distal third of the wrist was 0.4–0.6 SD lower than in the lumbar spine and femoral neck sites [31]. Perhaps this parameter has the greatest sensitivity in predicting the risk of fractures.

The frequency of OFF over 3 years in women with DM2 aged 55–84 years in our study was 3.0%. Similar data were obtained by Yalochkina et al., 2016, among 214 individuals with DM2 (44–88 years old); 5.1% of patients had OFF [32].

The investigations of association between osteoporotic fractures and risk factors for NCD are actively carried out in international and Russian studies. According to the multivariate analysis in our study, the odds of forearm fractures among postmenopausal women independently increased in those with past smoking by 2.23 times, in those with elevated level of total cholesterol higher than 200 mg/dL by two times, and the chance of a fracture was inversely related to BMI value.

Tobacco is the most common risk factor for osteoporosis. In a meta-analysis of 48 studies [33], bone density in smoking women decreased by about 2% for every 10 years, with a difference of 6% at the age of 80 years. In another meta-analysis [34], Kanis JA et al., 2005, showed that smoking is associated with an increased risk of any fracture compared to non-smokers both in men and women. Smoking in the past was associated with a significantly increased risk of fracture compared to non-smokers, but the risk was lower than that of current smokers. In addition, those who quit smoking had a lower risk of fracture than those who continued to smoke, but higher than those who had never smoked [34].

In our study, the risk of fractures increased in those women who smoked in the past, but we did not find an association between OFF and present smoking. We studied a population sample of postmenopausal women and the frequency of smoking in elderly women is quite rare in Russia. Therefore, it is possible the association between current smoking and OFF was not identified due to small numbers of smokers in our sample. The study limitation is that we have no data available on the duration and intensity of smoking. It might be that those who quit smoking had higher duration and intensity of smoking and/or had greater health problems as a result of which they quit smoking, compared to those who continue to smoke.

In general, possible mechanism by which smoking can increase bone loss is age at smoking initiation and its effect on body weight. Some studies have shown that initiation of smoking at age 13 affected bone accrual and was associated with low mean BMD at age 17 [35,36]. Thus, BMD deficiency at a young age led to a more rapid decrease in BMD in those over 50. By middle age, smokers weigh an average of 7–8 pounds less than nonsmokers [37]. Another study found quitting smoking significantly associated with increased body weight, fat, muscles, and functional mass that affected BMD [38]. Smoking cessation is significantly associated with increased body weight, fat, which ambiguously affects BMD and these mechanisms require further study.

The results of assessment of correlation between osteoporotic fractures and total cholesterol and BMD are contradictory. The effect of total cholesterol and its metabolites on osteoblastic activity was shown in vivo and in vitro [39]. Luegmayr E., et al., have shown that elevated plasma total cholesterol levels may decrease BMD and increase the incidence of fractures. In in vitro study, total cholesterol prolonged the survival of osteoclast-like cells that contribute to osteoporosis [40]. In addition, it was shown that a high level of total cholesterol was associated with a low level of 25 (OH) D, which was necessary for calcium absorption. [41,42]. In recent meta-analysis of Y.-Y. Chen et al., 2018, has shown that serum levels of total cholesterol are higher in women with postmenopausal osteoporosis than in the group with normal bone density. These data are consistent with the results in our study [43].

In our study, in unadjusted analysis, BMI in women with fractures was significantly lower than in those without fractures. However, after controlling for covariates, the risk of fractures was inversely related to BMI. Obesity is traditionally perceived as a protective effect on bone. This might be explained by an increase in the mechanical load on the skeleton and the ability of adipocytes to convert androgens to 17β-estradiol, which increases BMD [44].

The OFF is the most common type of fracture, and even a slight increase in the number of fracture cases significantly affects the need for health resources, especially among people of working age, when patients are to be “on sick leave” for 5–12 weeks or more depending on their profession. The NORA study has demonstrated that OFF at the age of ≥45 years increases the future risk of fracture of the proximal femur by 1.9 times [45]. These facts allow discussion about the “osteoporotic cascade” of fractures, when the next, and sometimes a series of new fractures occur after one fracture. Bone Health Alliance National Working Group experts note that OFF are characterized as osteoporotic fractures if there is concomitant osteopenia or osteoporosis by measuring BMD (T-score less than −1.0 SD) at the level of the lumbar spine or femur [46]. This allows us to suggest a shift of a paradigm shift from hip fractures to wrist fractures, and given the data from our study in Novosibirsk population, we could suppose the rationale for modification of criteria for osteoporosis diagnosis for the National Expert Working Group (USA).

However, current Russian recommendations on osteoporosis, 2017 [9] and the recommendations of the National Osteoporosis Foundation of the United States, 2014 [13], do not consider wrist fractures (in patients without preliminary hip/vertebral fractures or with BMD in the range of osteoporosis values) as an indication for pharmacotherapy [9,13].

Thus, there is currently no consensus among specialized bone research societies regarding whether low-energy wrist fractures should be considered as a criterion for diagnosing osteoporosis. Therefore, the identification and monitoring of these patients as a high-risk group for fractures is one of the important tasks of public health.

## 5. Conclusions

In the studied Siberian population sample of postmenopausal women aged 55–80 years old, the frequency of osteoporotic forearm fractures during the last 3 years was 3.9%. The cross-sectional determinants of OFF were smoking in the past and high total cholesterol value; body mass index value was inversely related to the risk osteoporotic fractures. The identification of mutual risk factors suggests deeper relationships between NCD and osteoporotic fracture development. The obtained data might have implications for fracture prevention in postmenopausal women.

## Appendix A

**Table A1:** Characteristics in groups of postmenopausal women aged 55–80 years with a history of OFF over the past 3 years and without a history of OFF.

Characteristics	OFF + n = 79	OFF − n = 1926	*p*
Age, years	68.8 ± 6.3	69.2 ± 6.8	0.611
Height, cm	157.7 ± 7.1	157.2 ± 6.1	0.453
Weight, kg	70.9 ± 13.2	75.9 ± 14.7	0.003
BMI, kg/m^2^	28.5 ± 4.7	30.7 ± 5.7	0.001
Waist circumference, cm	89.7 ± 11.9	95.2 ± 12.4	0.001
Hip circumference, cm	105.9 ± 10.3	109.1 ± 10.7	0.013
Waist circumference/Hip circumference	0.85 ± 0.6	0.87 ± 0.7	0.001
SBP, mmHg	143.4 ± 20.9	144.9 ± 21.6	0.528
DBP, mmHg	83.4 ± 10.1	82.3 ± 10.8	0.362
FBG, mmol/l	6.3 ± 5.1	6.3 ± 1.8	0.937
Total cholesterol, mg/dL	225.9 ± 37.1	219.1 ± 46.7	0.201
TG, mg/dL	133.5 ± 103.1	136.8 ± 84.1	0.739
HDL-C, mg/dL	53.2 ± 14.1	53.0 ± 14.9	0.918
PF10, n/%			
>75‰	24/30.4%	732/38%	0.171
≤75‰	55/69.6%	1194/62%	0.171
Education (n/%)			
Univer education	28/35.4%	580/30.1%	0.313
Secondary education	48/60.8%	1214/63.0%	0.682
Primary education	3/3.8%	132/6.9%	0.288
DM2 (n/%)	13/16.5%	423/22.0%	0.245
Smoking, n (%)			
Smoking present n (%)	3/3.9%	90/4.6%	0.717
Smoking in the past n (%)	8/10.3%	107/5.6%	0.087
Non smoking	67/85.9%	1727/89.8%	0.168
Postmenopause duration, years	19.5 ± 7.9	19.7 ± 8.4	0.834

The values are presented as M±SD or n/%
